# A multi-scale feature fusion neural network for multi-class disease classification on the maize leaf images

**DOI:** 10.1016/j.heliyon.2024.e28264

**Published:** 2024-03-20

**Authors:** Liangliang Liu, Shixin Qiao, Jing Chang, Weiwei Ding, Cifu Xu, Jiamin Gu, Tong Sun, Hongbo Qiao

**Affiliations:** a College of Information and Management Science, Henan Agricultural University, Zhengzhou, Henan 450046, PR China; b College of Agriculture, Shihezi University, Shihezi, Xinjiang 832061, PR China

**Keywords:** Multi-scale, Residual network, Multi-class, Maize leaf images

## Abstract

Maize is a globally important cereal crop, however, maize leaf disease is one of the most common and devastating diseases that afflict it. Artificial intelligence methods face challenges in identifying and classifying maize leaf diseases due to variations in image quality, similarity among diseases, disease severity, limited dataset availability, and limited interpretability. To address these challenges, we propose a residual-based multi-scale network (MResNet) for classifying multi-type maize leaf diseases from maize images. MResNet consists of two residual subnets with different scales, enabling the model to detect diseases in maize leaf images at different scales. We further utilize a hybrid feature weight optimization method to optimize and fuse the feature mapping weights of two subnets. We validate MResNet on a maize leaf diseases dataset. MResNet achieves 97.45% accuracy. The performance of MResNet surpasses other state-of-the-art methods. Various experiments and two additional datasets confirm the generalization performance of our model. Furthermore, thermodynamic diagram analysis increases the interpretability of the model. This study provides technical support for the disease classification of agricultural plants.

## 1 Introduction

Maize is a significant cereal crops, which provides a vital source of nutrition for millions of people globally [1]. Efficient irrigation and improved nutrition management can increase crop yields [2]. However, the crop's yield is vulnerable to numerous biotic and abiotic factors, including various plant diseases [3], [4]. In particular, the pests and diseases risk reduce achievable yields by more than 50% [5]. One of the most common and destructive diseases that affect maize is maize leaf disease. Maize leaf disease is caused by stenocarpella maydis, which infects the leaves and stalks of maize plants [6]. Symptoms of maize leaf disease can include brown lesions or streaks on the leaves, which may develop into elongated spots that can cover the entire leaf, causing it to wither and die. This disease can ultimately affect the entire plant, leading to a significant reduction. Therefore, adopting innovative ways to improve maize disease detection in adverse conditions is crucial for agricultural yield [7].

Three leaf diseases, including common_rust, gray_spot, and blight, are among the most prevalent significant diseases that affect maize crops [8], [9]. In theory, these diseases are easy to diagnose. For example, common rust causes rusty brown pustules, while gray spot causes rectangular lesions with a yellow halo [10]. Blight causes irregularly-shaped lesions that may have a water-soaked appearance, and it can also affect the stem and other parts of the plant [1]. However, distinguishing and identifying these similar diseases in farmland requires both professional knowledge and a lot of energy and resources. Previously, farmers had to send contaminated leaves to pathology laboratories, where pathologists confirmed the condition, which proved to be a time-consuming task. Therefore, achieving automation of disease detection systems to accelerate crop diagnosis is crucial. However, how to automatically and accurately identify maize leaf diseases remains a key research topic in current crop disease research.

## 2 Related work

In recent years, there has been a growing trend of using artificial intelligence (AI) methods for disease detection and management [11], [12], [13]. One approach involves utilizing deep learning algorithm to analyze images of plants and identify disease symptoms [14], [15]. Convolutional neural networks (CNNs) have been successfully applied to image analysis tasks and have achieved high accuracy in disease detection [16], [17]. For example, Hassan et al. [18] developed a depth separable CNN to classify healthy and diseased leaves of 14 different plant species. While Naik et al. [19] developed a CNN model named SECNN for detecting various types of chilli leaf disease from digital camera images. In addition, a Faster R-CNN was used for detecting diseases from rice leaf [20]. However, it can be challenging to develop accurate CNN models for diseases like maize leaf disease, as large and diverse datasets for training may not be readily available.

To address the challenge of dealing with limited sample sizes, transfer learning emerged as a powerful solution [21]. This method has been trained to recognize similar objects, and then adapting them for specific tasks. In situations where extensive training datasets are not readily accessible, transfer learning has proven to be a reliable technique for enhancing the performance of deep learning models. Notably, it often involves the utilization of pre-trained weights from well-established sources like the ImageNet dataset [22]. Numerous studies have substantiated the effectiveness of transfer learning in the context of plant disease identification [23], [24]. Paymode et al. introduced a transfer learning approach based on the VGG architecture, specifically designed for the early forecasting of grape and tomato leaf diseases [25]. Thangaraj et al. proposed a transfer learning-driven deep CNN to identify tomato leaf diseases [26]. Additionally, Krishnamoorthy et al. harnessed the power of the InceptionResNetV2 network to effectively recognize diseases in rice leaf images [27]. It is important to note that while transfer learning offered remarkable advantages, fine-tuning pre-trained models to detect specific disease symptoms necessitates a nuanced understanding of the particular disease domain. Furthermore, this process may demand substantial computational resources. Thus, successful implementation of transfer learning in disease recognition demands a combination of technical expertise and computational capacity.

Moreover, the exploration of alternative methods for detecting maize leaf diseases has led to the consideration of multi-spectral and hyper-spectral imaging [28], [29]. These advanced techniques involve capturing images of maize leaves at various wavelengths and subsequently analyzing these images to identify disease symptoms. To overcome the challenge of selecting critical raw bands from hyper-spectral images, Park et al. introduced the application of minimum redundancy and maximum relevance feature selection techniques [30]. This approach had been successfully employed in classifying various apple leaf conditions, showcasing its potential in disease detection. Furthermore, Pallathadka et al. developed an integrated framework that combined Support Vector Machines (SVM), Naive Bayes, and CNN to automatically classify and assessed the severity of crop leaf diseases by using hyper-spectral images [31]. This comprehensive approach demonstrated promising results in disease identification. However, multi-spectral and hyper-spectral imaging techniques comes with associated costs, which can be relatively high. Additionally, interpreting data obtained through these techniques requires a high level of expertise in image processing and data analysis. Therefore, while these methods offered valuable alternatives for disease detection, they also posed practical challenges that need to be addressed, including cost considerations and the need for specialized knowledge.

In summary, although AI methods have showcased considerable promise in the realm of maize leaf disease detection, there exist notable obstacles that demand careful consideration to elevate the accuracy and efficacy of these methods when applied in practical. These challenges encompass: image quality, resemblance to other diseases, variations in disease severity, insufficient dataset availability, and restricted interpretability. Tackling these challenges requires ongoing research, innovation, and collaboration across domains, including computer vision, agriculture, and machine learning. Overcoming these obstacles is pivotal in fully realizing the potential of AI for timely and accurate maize leaf disease detection, thereby contributing to the advancement of agriculture and food security.

In this study, we develop a model for detecting the maize leaf diseases from leaf images that named MResNet. The main contributions are listed as follows:

(1) We propose a multi-scale residual net (MResNet) for classifying multi-type maize leaf diseases from maize images.

(2) A hybrid feature weight optimization method is used to optimize and fuse the feature mapping weights of two subnets.

(3) We use Class Activation Mapping (CAM) visualization methods to conduct interpretability analysis of model concerns.

(4) Sufficient experiments and additional datasets have verified the generalization performance of our model.

## 3 Materials and methods

### 3.1 Data set

**Figure 1:**
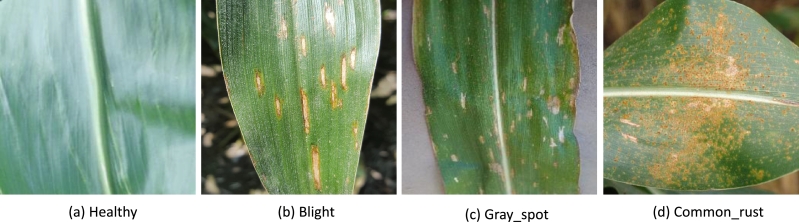
Visualization of four types of corn or maize leaves.(a) is healthy leaf, (b) is blight leaf, (c) is gray_spot leaf, and (d) is common_rust leaf.

Maize leaf disease datasets typically consist of a collection of corn or maize leave images affected by various diseases, including maize leaf diseases. In our study, our samples are from PlantVillage^1^ and PlantDoc datasets^2^ [32]. PlantVillage is a large plant image dataset, which were collected from plantations and gardens. It contains over 54,000 images of diseased and healthy leaves, stems, and fruits of plants, along with information about the plant species and the type of disease present in the image. PlantDoc is a plant image dataset, which specifically focus on crop diseases. It contains over 87,000 images of crops affected by different diseases, such as plant species, disease type, and geographic location. We selected images related to corn or maize leaf diseases as a sample and removed that are not useful to our research goal. Finally, we build a corn or maize leaf disease dataset (CMLset) which consisted of 4188 images and their label with four types, including common rust samples (1306 images), gray spot samples (574 images), blight samples (1146 images), and healthy samples (1162images). Visualization of four types of samples is shown in Fig. 1. All selected samples are shown in Table 1.

**Table 1:** Details of corn or maize leaf image dataset utilized in our study.

KL Grade	Type	Original number	Enhanced number
0	Common_rust	1306	5224
1	Gray_spot	574	2296
2	Blight	1146	4584
3	Healthy	1162	4648

### 3.2 Data preprocessing

The challenges in maize leaf image disease classification have been introduced in the previous Section 3.1, including non-uniform size, complex background, inconspicuous lesions, and high similarity of lesions. We adopted the following image preprocessing strategies to address the above issues: (1) Non-uniform size: Maize leaves vary in size, shape, and orientation, making it difficult to compare features between different leaves. To address this challenge, we adopted cropping and resizing methods to standardize the size and orientation of the images (256×256). (2) Complex background: Maize leave images usually contain complex backgrounds, such as soil, grass, and other vegetation, which interfere with disease classification algorithms. To address this challenge, image segmentation techniques was used to isolate the leaf from the background. (3) Inconspicuous lesions: The symptoms of maize leaf disease can be inconspicuous and difficult to detect. To address this challenge, image enhancement techniques such as contrast adjustment and sharpening methods were used to enhance the visibility of disease symptoms in the images. (4) High similarity of lesions: The lesions caused by the maize leaf disease are similar to those caused by other diseases, making it difficult to accurately identify the specific disease affecting the leaf. To address this challenge, machine learning algorithms were trained on a diverse set of images of leaves affected by different diseases to improve their ability to distinguish between different types of lesions. (5) Light issues: The lighting and contrast play an important role in plants disease detection algorithms. We used image normalization to adjust the image intensities to account for these differences. This technique can improve the accuracy and consistency of disease detection by ensuring that disease symptoms are accurately represented in the images. (6) The limited number of images: To alleviate this problem, we applied various image augmentation techniques to the training images, including random cropping, flipping, rotation, and brightness adjustments.

The details of the reprocessed maize leaf image dataset are shown in Table 1. Specifically, the processed type common_rust has 5224 images, type gray_spot has 2296 images, type blight has 4584 images, and healthy has 4648 images.

### 3.3 Overview of our method

**Figure 2:**
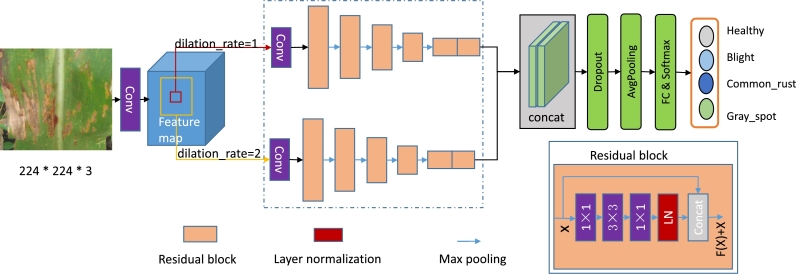
The pipeline for the proposed MResNet.

As shown in Fig. 2, we present MResNet, a novel multi-scale residual neural network, for classifying multi-type of maize leaf diseases from maize images. MResNet comprises two residual subnets that operate with different scales, enabling the model to detect diseases from maize leaf images at multiple scales. In residual subnets, we used residual blocks to capture multi-scale features and enhance the accuracy of disease detection. The residual block can obtain more abundant features from the image while optimizing the model structure. We also proposed a hybrid feature weight optimization method for two subnet features fusion, which leaded to improved performance and more accurate disease detection.

### 3.4 Multi-receptive-field strategy

To solve the problem of insufficient sample size and improve the richness of contextual semantic feature extraction, we used multi-scale subnets to help the model obtain more contextual semantic information. Specifically, when images were input into the model, multiple receptive fields were used to obtain contextual semantic information of different scales and then sent them to different residual subnets. The residual subnet was a key component of MResNet. As shown in Fig. 2, the residual subnet was composed of convolutional layers and encoder blocks. In detail, a residual subnet composed of 10 convolution layers, 4 residual units, 4 concatenate layers, and some batch normalization layers (BN), leaky rectified linear units (LReLU). Each residual subnet can obtain different scale feature maps from the dilated convolution. This means that the semantic relationship of two subnets was different. Both subnets obtained different semantic information from the same input image, and the back propagation method was used to update the semantic information of pixels during model training.

Let *x* be an input feature map, and set the dilated convolution with k×k kernel size and the dilated rate is *r*. The output feature map of *i*-th dilated convolution set to $x_{i}$, $x_{i}$ also denotes the input feature map of the *i*-th residual subnet. We define the output of the *i*-th residual subnet as $y_{i}$, which is defined in Eq. (1):

(1) $$y_{i}=\sum_{k=1}^{m}x_{i}^{r\times k}w[k],$$

where w[k] is a filter matrix and *m* is the length of w[k].

There are *n* residual blocks in a residual subnet. The input of the (l+1)-th residual block in the *j*-th residual subnet is defined in Eq. (2):

(2) $$R_{j}^{l+1}=R_{j}^{l}+F(R_{j}^{l},W_{j}^{l}),$$

where $R_{j}^{l+1}$ is the input of the *l*-th residual block in the *j*-th residual subnet, $R_{j}^{l}$ is the output, $W_{j}^{l}$ is the convolutional weight. F() is the residual function.

### 3.5 Fusion method in residual network

The concat() method is the concatenation operation. concat() method is the combination of channel numbers, which means that the number of features (channels) describing the image itself has increased, while the information under each feature has not increased. The definition concat() is as follows:

(3) $$Out_{concat}=concat(R_{1},R_{2},...,R_{j},...,R_{n}),$$

where $R_{j}$ is the output feature map of the *j*-th residual subnet (j∈[1,n]). It fuses features from multiple convolutional feature extraction networks.

The output of concat() method is the combination of channel numbers. The convolution operation is defined as ⁎ and the *j*-th feature map is $R_{j}$. $A_{i}^{1}$, $A_{j}^{2}$ are the channels of $R_{j}$,..., $A_{j}^{c}$. Then Eq. (3) can be redefined as:

(4) $$Out_{concat}=\sum_{i=1}^{c}A_{1}^{i}⁎K_{1}^{i}+...+\sum_{i=1}^{c}A_{n}^{i}⁎K_{n}^{i+(n-1)⁎c},$$

where *n* is the number of feature maps. *K* and *c* are the convolution kernel and the number of channels. In our study, *n* is set to 2.

### 3.6 Fusion method of subnets

As one of the contributions of MResNet, we propose a feature fusion strategy to optimize the weights of two subnets. Let $f_{net1}$ and $f_{net2}$ be the feature maps of two subnets, respectively. $O_{net1}$ and $O_{net2}$ are the output feature of two residual subnets. Let O(f) be the output of the concatenation operation, which is defined in Eq. (5):

(5) $$O(f)=concat(\theta O_{net1},(1-\theta )O_{net2}),$$

where *θ* is the weight parameter.

An output of a subnet consists of several channels. Based on Eq. (4) and Eq. (5), we redefined O(f) that is shown in Eq. (6):

(6) $$O(f)=\theta \sum_{j=1}^{c}A_{1}^{j}⁎K_{1}^{j}+(1-\theta )\sum_{j=1}^{c}A_{2}^{j}⁎K_{2}^{j+(n-1)⁎c}.$$

## 4 Experiments and results

### 4.1 Experimental settings

The richness of features obtained by the model is positively correlated with the number of residual subnets. However, the number of residual subnets is limited by computer server hardware. In our study, we made a trade-off between the structure of our proposed method. Ultimately, we chose to use 2 encoder networks (residual subnets) with dilation rates of 1 and 2. To unify the image size, we used resize() in the Skimage package [33] to unify the input image size (256×256). All samples were divided into training and testing sets with radio 8:2. We used an image flip method to augment training data. In addition, the accuracy (Acc), precision (Prec), recall (Rec), and $F_{1}$-score work as the main metrics. The project and initial parameters are shown in Table 2.

**Table 2:** Computer and tool configuration.

Project and initial parameters	Content
System	Linux
Framework	Tensorflow
CPU	Xeon e2670
GPU	NVIDIA GeForce RTX 3090
RAM	16G
epoch	200
mini-batch size	3
learning rate	0.001
drop-out rate	0.3
loss function	Adam optimizer [34]

### 4.2 Comparison results

We compared the performance of MResNet with the methods which not based on a transfer learning strategy (SqueezeNet [35], VGG16 [36], ResNet101 [37], CNNet [38], and eNet [39]) and the methods which are based on a transfer learning strategy (InceptionV3 [40], VGG16, and ResNet18 [41]). The comparison results were shown in Table 3. Among these methods, our proposed method achieved the best performance, accuracy: 97.45%, precision: 97.27%, recall: 97.04%, and $F_{1}$-score: 97.83%. In addition, there were two issues need to pay attention to in these comparison methods. (1) For non-transfer learning methods, the depth of the network model was proportional to the performance rate. For a neural network, as the depth increased, the performance improved. For example, SqueezeNet was a simple 8-layer CNN, while VGG16 has more layers than SqueezeNet, and the overall performance of the VGG16 model was higher than SqueezeNet. ResNet101, CNNet, and eNet used residual mechanism, dense mechanism, and multi-strategy integration method to build neural networks with more layers. These three methods achieved a better performance than VGG16. In these non-transfer learning methods, these deeper models achieved better performance. (2) The performance of the transfer learning-based methods achieved better than that of the method without a transfer learning strategy. Although increasing the depth could improve the performance of the model, this strategy was trapped in the sample size and model structure, which usually caused prone to over-fitting, gradient disappearance, and other problems. The emergence of transfer learning alleviates the above problems. For the same method or structurally similar methods VGG16 & VGG16 (TL), ResNet101 & ResNet18 (TL). The accuracy of the transfer learning-based methods increased by 2.97% and 5.76%, respectively. Although ResNet101 was deeper than ResNet18 (TL).

**Table 3:** Comparison results of MResNet and state-of-the-art methods. Transfer learning(TL).

Method	Acc (%)	Prec (%)	Rec (%)	F1-Score (%)
SqueezeNet	79.39	77.18	75.43	78.75
VGG16	87.05	87.95	88.34	87.28
ResNet101	90.44	90.42	91.37	91.58
CNNet	92.63	91.44	92.63	91.95
eNet	95.00	94.00	94.00	95.00
InceptionV3(TL)	72.22	73.21	75.89	75.87
VGG16(TL)	90.02	90.18	91.43	90.75
ResNet18(TL)	96.20	97.05	96.13	96.47
MResNet	97.45	97.27	97.04	97.83

It should be noted that our proposed method did not utilize transfer learning strategy, yet it outperformed all the other methods. This was mainly due to our use of a multi-scale residual subnets that obtained more semantic information from the same images, as well as our novel feature fusion strategy that optimized the feature map of two subnets. To further demonstrate the effectiveness of these strategies, we conducted ablation experiments.

### 4.3 Ablation experiments

We conducted the ablation experiments to verify the role of components in MResNet. Specifically, ResNet: A classification model that retained only one residual branch. ResNet(TL): A classification model that was based on transfer learning and residual branch. MResNet1: Only kept one branch with dilation rates of 1 in MResNet. MResNet2: Only kept one branch with dilation rates of 2 in MResNet. MResNet(add): Preserved two residual networks and used the “add” function to fuse feature maps. MResNet: Our proposed model used the “concat” function to fuse the feature maps of two residual subnets.

The results were shown in Table 4, ResNet, MResNet1, and MResNet2 achieved the worst classification performance among these four methods. Although the residual strategy could reduce the loss of semantic information and provided more semantic information for the model. However, compared to the other three methods, the effectiveness of this strategy was limited. However, ResNet(TL) could improve the performance of the model by adopting the transfer learning strategy, while MResNet(add) and MResNet adopted the dual branches to obtain more semantic information. These strategies help these models obtain more contextual semantic information than ResNet, which will be beneficial to improving the performance of the model. The performance of transfer learning-based method (ResNet(TL)) exceeded that of MResNet(add). However, the evaluation indicators such as MResNet(add) and ResNet(TL) accuracy and accuracy were very close, which meaned that multi-scale strategies were also an effective method. In the end, MResNet replaced the fusion method of two subnet features in MResNet(add), achieving the best classification results. This was mainly because using concatenation functions to fuse features could eliminate noise in the features.

**Table 4:** Results of ablation experiments.

Method	Acc (%)	Prec (%)	Rec (%)	F1-Score (%)
ResNet	89.67	89.53	90.02	90.13
ResNet(TL)	96.20	97.05	96.13	96.47
MResNet1	89.72	89.60	90.13	90.16
MResNet2	90.06	90.37	90.25	90.43
MResNet(add)	95.64	95.37	95.62	95.70
MResNet	97.45	97.27	97.04	97.83

### 4.4 Impact of fusion method

To verify the performance of the fusion strategy pair of two residual subnets, we evaluated the value of the fusion weight parameter *θ* (θ∈[0,1]). We set the *θ* values to 0, 0.2, 0.4, 0.6, 0.7, 0.8, and 1 respectively to verify the performance of MResNet. The results were shown in Table 5. When *θ* was 0 or 1, MResNet degenerated into different residual subnets (MResNet1 and MResNet2 in Table 4), resulting in the worst performance of the model. As the value of *θ* increases, the performance of MResNet gradually improved, and when θ=0.7, the model achieved its best performance. Subsequently, the performance of the model gradually decreased. Finally, we chose θ=0.7 as the parameter for MResNet.

**Table 5:** Impact of fusion method on MResNet.

*θ*	Acc (%)	Prec (%)	Rec (%)	F1-Score (%)
0	89.72	89.60	90.13	90.16
0.2	92.71	92.37	92.23	93.01
0.4	94.29	94.82	94.57	94.76
0.6	96.87	96.92	96.73	96.68
0.7	97.45	97.27	97.04	97.83
0.8	94.45	95.01	94.38	95.02
1	90.06	90.37	90.25	90.43

### 4.5 Visualization analysis

**Figure 3:**
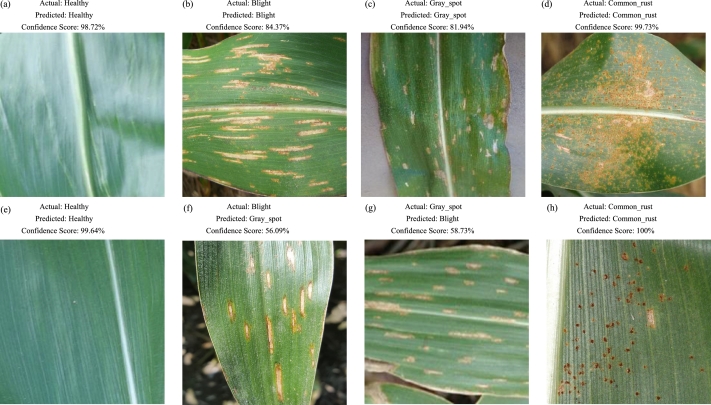
Visualization of MResNet.

To further validate the performance of MResNet, we selected two samples of each type and present the prediction results, as shown in Fig. 3. For each type of sample, we selected two groups for display. Each image was annotated with the corresponding real label, predicted label, and confidence score. As shown in Fig. 3, our model had the highest correct recognition rate for common_rust-type leaves, as the lesion areas above this type of leaves were dark in color and exhibit patchy distribution, which had extremely obvious characteristics compared to the other three types of leaves. Secondly, our model also had a relatively high confidence score (greater than 98%) for predicting healthy-type leaves. This was because the healthy-type leaf page was not as smooth as the lesion, but some leaves with less severe lesions also exhibited the same features in most areas as health-type leaves, which might affect the recognition accuracy of the model. Also, for gray_spot, the recognition accuracy of a spot was relatively low, mainly due to the problem of imbalanced samples. This type of sample only had 574 pieces, while the sample size of the other three types exceeded 100 pieces. Finally, the model achieved the lowest recognition for samples of type Blight. Usually, Blight samples were mistakenly identified as gray_spot Sample of type spot. The main reason was that the morphology of spot type leaf lesion areas was between Blight and gray_spot between samples of type spot. As shown in Fig. 3, gray_spot The lesion area of spot type samples was closer to Blight, so the model mistakenly took a portion of gray_spot as the Blight type.

### 4.6 Generalization verification

To verify the generalization performance of the model, we conducted the experimental validation of the MResNet method on citrus leaves dataset (CLset) and citrus fruits dataset (CFset) [42]. The citrus leaf dataset comprised 609 samples with five classes (Healthy:58, BlackSpot:171, Canker:163, Greening:204, and Melanose:13). The citrus fruits dataset comprised 150 samples with five classes (Healthy:22, BlackSpot:19, Canker:78, Greening:16, and Scab:15).

We used the weights trained by the MResNet on CMLset and modified the output layer of the model based on additional datasets. The experimental results were shown in Table 6. Our proposed MResNet achieved the best classification performance on both additional datasets. This means that our model had good generalization performance. Notably, MResNet performs better on CLset compared to CFset, and there were two primary reasons for this difference. Firstly, the heterogeneity of samples played a significant role. Both CMLset and CLset comprise crop leaf diseases and pests, resulting in similarities among their samples. In contrast, CMLset and CFset exhibited heterogeneity. Secondly, the smaller sample size of CFset might also influence the model's performance.

**Table 6:** Comparison results on citrus leaves dataset and citrus fruits dataset. Transfer learning(TL).

Method	Citrus leaves dataset				Citrus fruits dataset			
	Acc (%)	Prec (%)	Rec (%)	F1-Score (%)	Acc (%)	Prec (%)	Rec (%)	F1-Score (%)
SqueezeNet	72.08	71.94	72.13	72.40	68.70	68.52	68.72	68.84
VGG16	85.72	85.48	86.01	85.48	74.08	74.52	74.46	74.73
ResNet101	88.96	89.03	88.72	88.73	77.42	77.49	77.50	77.62
CNNet	90.40	90.29	90.37	90.52	80.67	81.06	80.78	80.67
eNet	94.16	94.47	93.89	94.72	85.49	85.78	85.64	85.92
InceptionV3(TL)	83.28	83.08	83.75	83.40	81.45	81.40	81.67	81.80
VGG16(TL)	92.71	92.08	92.94	92.57	90.26	90.31	90.18	90.60
ResNet18(TL)	97.43	97.57	97.62	97.80	92.37	92.74	92.15	92.34
MResNet	98.03	97.85	97.73	98.00	94.09	94.28	94.72	94.00

## 5 Discussion

In this study, we proposed a multi-scale residual net (MResNet) for classifying multi-type maize leaf diseases from maize images. We utilized a hybrid feature weight optimization method to optimize and fused the weights of two subnets. MResNet was validated on a maize leaf diseases dataset. MResNet achieves 97.45% accuracy. The performance of MResNet surpassed other state-of-the-art methods.

### 5.1 Loss and accuracy plots of MResNet

**Figure 4:**
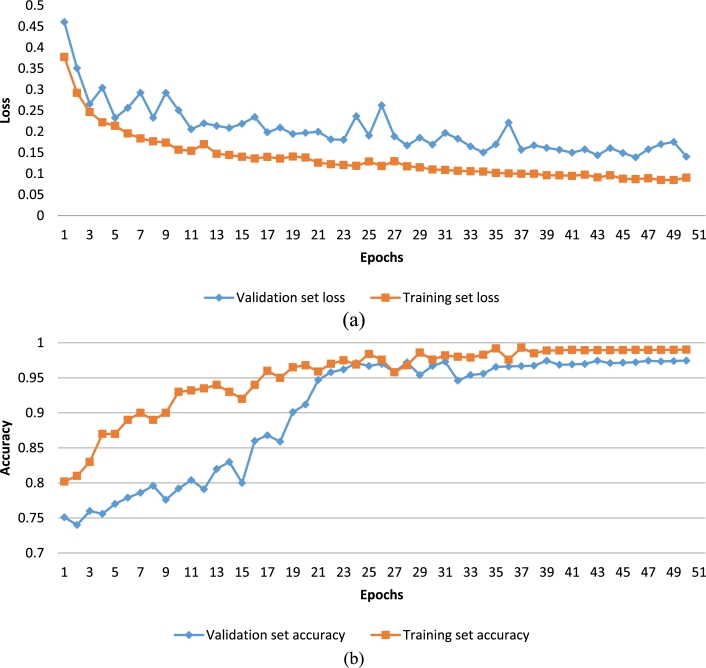
The training and validation loss and accuracy plots of MResNet.

To prevent over-fitting problems, we used the early-stopping strategy during the training process. Early-stopping is to stop training when the performance of the model on the verification set no longer increases, so as to achieve the role of full training and avoid over-fitting. In Fig. 4, we show the training and validation loss graph (Fig. 4(a)) and accuracy graph (Fig. 4(b)) generated by MResNet. The results are based on BCE loss function.

In Fig. 4(a) and Fig. 4(b), MResNet stopped before 50 epochs in the training process. In detail, the loss value of the model decreases significantly with the beginning of training, indicating an appropriate learning rate and gradient descent process. Subsequently, the loss curve stabilized, as shown in Fig. 4(a), and the change in the loss value of the model was not as significant as at the beginning. After approximately 40 periods, the model gradually converged. To prevent over-fitting, terminate model was trained based on patient values before 50 epochs. This means that our training strategy effectively avoided the over-fitting and under-fitting problems in the model training process. Finally, we saved the optimal weights for the MResNet training phase.

### 5.2 Visualization of CAM from MResNet

**Figure 5:**
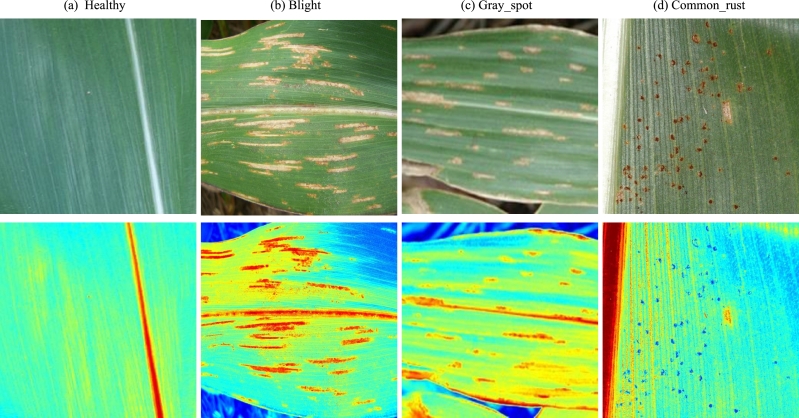
Visualization of CAM from MResNet.

Furthermore, to increase the interpretability of the model, we used CAM to display thermodynamic diagrams on 4 typical images. All CAM visualizations came from the last convolutional layer of MResNet. Fig. 5 represented four types of samples: healthy, blight, gray_spot, and common_rust. From the thermodynamic diagram, it could be seen that the model had no obvious focus in the thermodynamic diagram (Fig. 5) of the healthy sample. And the model had obvious concerns about the remaining three diseased leaves. According to the different types of diseased leaves, the heat map focused of blight, gray_spot samples exhibit a point like distribution, which also increased the difficulty for the model to distinguish between these two types of samples. This had been proven in Fig. 3. In addition, the heat map of the common_rust sample shown a blocky distribution of interest points, which was clearly distinguished from the other two diseased samples.

**Figure 6:**
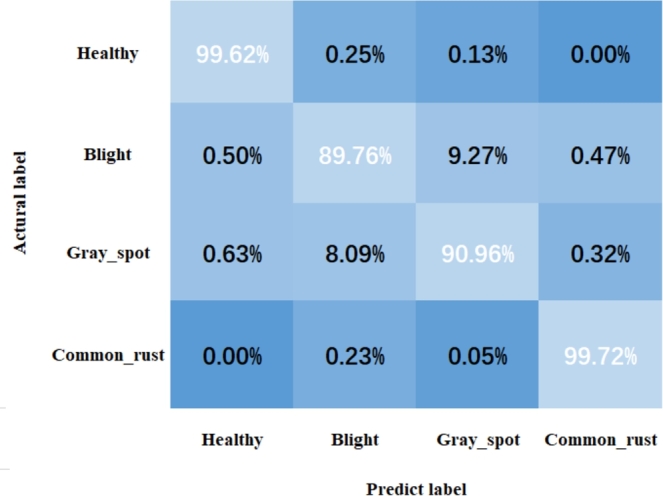
Confusion matrix visualizes the performance of MResNet.

Furthermore, we used confusion matrix to show the classification effect of our proposed MResNet in subclasses of CMLset dataset. As shown in Fig. 6. MResNet could accurately identify health and common_rust samples (accuracy: 99.62% and 99.72%). In contrast, the recognition accuracy of MResNet for light and gray_spot samples had decreased significantly, mainly because these two leaf diseases were difficult to distinguish, which had been visually verified in the segmentation results in Fig. 5.

## 6 Conclusions

In this study, we proposed a multi-scale residual net (MResNet) for classifying multi-type maize leaf diseases from images. MResNet consisted of two residual subnets at different scales, which allowed the model to detect diseases from maize leaf images at different scales. These multi-scale residual subnets used residual blocks to improve the performance of deep neural networks. Furthermore, we introduced a hybrid feature weight optimization method to optimize and fuse the weights of two subnets. We also validated the model's generalization performance on two additional datasets. In future research, we plan to focus on the classification task of similar diseases in crops.

## CRediT authorship contribution statement

**Liangliang Liu:** Methodology, Investigation, Conceptualization. **Shixin Qiao:** Investigation, Funding acquisition, Conceptualization. **Jing Chang:** Software, Data curation. **Weiwei Ding:** Validation, Formal analysis, Data curation. **Cifu Xu:** Data curation. **Jiamin Gu:** Software. **Tong Sun:** Resources, Methodology, Data curation. **Hongbo Qiao:** Investigation, Funding acquisition, Conceptualization.

## Declaration of Competing Interest

The authors declare that they have no known competing financial interests or personal relationships that could have appeared to influence the work reported in this paper.

## Data Availability

The dataset are from PlantVillage (https://www.kaggle.com/datasets/emmarex/plantdisease) and PlantDoc datasets (https://github.com/pratikkayal/PlantDoc-Dataset).

## Footnotes

1 https://www.kaggle.com/datasets/emmarex/plantdisease.

2 https://github.com/pratikkayal/PlantDoc-Dataset.
